# A comparison of blood pressure in community pharmacies with ambulatory, home and general practitioner office readings: systematic review and meta-analysis

**DOI:** 10.1097/HJH.0000000000001443

**Published:** 2017-06-07

**Authors:** Ali Albasri, Jack W. O'Sullivan, Nia W. Roberts, Suman Prinjha, Richard J. McManus, James P. Sheppard

**Affiliations:** a Nuffield Department of Primary Care Health Sciences, University of Oxford, Radcliffe Observatory Quarter; b Bodleian Health Care Libraries – Knowledge Centre Site, Knowledge Centre, Oxford, UK

**Keywords:** ambulatory, blood pressure, clinic, general practitioner, home, hypertension, pharmacy

## Abstract

**Background:**

Blood pressure (BP) readings are traditionally taken in a clinic setting, with treatment recommendations based on these measurements. The clinical interpretation of BP readings taken in community pharmacies is currently unclear. This study aimed to systematically review all literature comparing community pharmacy BP (CPBP) readings with ambulatory BP monitoring (ABPM), home BP monitoring and general practitioner clinic readings.

**Method:**

Studies were included if they compared CPBP with at least one other measurement modality used for the diagnosis or management of hypertension. Mean CPBP readings were compared with other measurement modalities and summarized using random-effects meta-analyses. The primary outcome was to compare CPBP with gold standard ABPM readings.

**Results:**

Searches generated 3815 studies of which eight were included in the meta-analyses. The mean systolic CPBP-daytime ABPM difference was small [+1.6 mmHg (95% confidence interval −1.2 to 4.3) three studies, *n* = 319]. CPBP was significantly higher than 24-h ABPM [+7.8 mmHg (95% confidence interval 1.5–14.1) three studies *n* = 429]. Comparisons with general practitioner clinic readings (six studies, *n* = 2100) were inconclusive with significant heterogeneity between studies. CPBP and home BP monitoring readings (five studies, *n* = 1848) were nonsignificantly different. Diastolic comparisons mirrored systolic comparisons in all but the CPBP-daytime ABPM comparison, where CPBP was significantly higher.

**Conclusion:**

Current evidence around the clinical interpretation of CPBP is inconclusive. Although this review suggests that adopting the 135/85 mmHg threshold for hypertension might be reasonable and potentially result in a higher sensitivity for detecting patients with truly raised BP in pharmacies, the impact of this lower threshold on increased referrals to general practice clinics must be considered.

## Abstract 2

Supplemental Digital Content is available in the text

## INTRODUCTION

Hypertension is the most common, preventable risk factor for cardiovascular events [1], with almost a third of all adults in England affected [2]. Hypertension-related appointments make up approximately one in 10 of all general practitioner consultations each year [3] and with the workload of general practitioners thought to be nearing saturation point [4]; alternative models of care are needed to ensure appropriate management of hypertension in the community.

National guidelines in the United Kingdom recommend out-of-office measurements, in the form of ambulatory blood pressure (BP) monitoring (ABPM), as the ‘gold standard’ for the diagnosis of hypertension [5]. Where ABPM is not possible, home BP monitoring (HBPM) is recommended, but even this is not acceptable to all patients with hypertension [6]. These measurement modalities have shown to be more closely associated with patient's future risk of cardiovascular events than clinic BP [7,8], and when combined with general practitioner clinic measurements, make up the foundation of hypertension diagnosis and monitoring. Thresholds of 140/90 (clinic) and 135/85 mmHg (daytime ABPM/HBPM) are widely accepted for hypertension diagnosis and management among nondiabetic adults under the age of 80 years [5,9].

Routine BP check-ups in pharmacies could save both general practitioner time and healthcare costs [5,11]. Almost 90% of the UK population live within a 20-min walk from a pharmacy [10] and more than 20 million people visit a pharmacy in Europe every day [11]. Furthermore, no appointment is generally required to be seen for a BP measurement [12]. However, it is unclear how community pharmacy BP (CPBP) measurements relate to those taken in a general practitioner clinic or out-of-office setting, and therefore how these readings should be used in the management of patients with hypertension. In the United Kingdom, BP guidance from the Royal Pharmaceutical Society suggests that pharmacists could consider referring a patient to their doctor if their BP is above 140/90 mmHg [13].

A literature review conducted in 2009 [14] identified three studies comparing CPBP with other measurement modalities. No quantitative analysis was possible due to the sparsity of data and methodological concerns regarding eligible articles. Subsequent studies have suggested that pharmacy BP readings may be comparable with out-of-office monitoring [15,16]. This study systematically reviewed all existing literature comparing BP readings taken in community pharmacies with ABPM, HBPM and general practitioner clinic readings.

## METHODS

### Search strategy

The current study was conducted and reported according to the Preferred Reporting Items for Systematic reviews and Meta-Analyses guidelines (PRISMA) [17]. The fulfilled PRISMA checklist can be found in Supplementary Appendix 1.

A literature search in the EMBASE, CINAHL and MEDLINE databases was conducted to identify studies which compared CPBP readings to those of ABPM, HBPM or general practitioner clinic readings. No language limit or study design filters were set. Searches were conducted on articles from January 2009 to December 2015 in MEDLINE and EMBASE (updating the original search from December 2009) [14] and from inception to December 2015 in CINAHL. Further studies were identified through searching references of full text articles screened and of the previous systematic review on the topic [14]. The search strategy can be found in Supplementary Appendix 2.

The study protocol was registered on PROSPERO: International Prospective Register of Systematic Reviews and can be found online (http://www.crd.york.ac.uk/prospero) – registration number CRD42016032518.

### Selection of studies and inclusion criteria

Titles, abstracts and full texts were independently reviewed by two members of the review team (A.A. and J.O.S.). Conflicts were resolved through discussion, or through involvement of a third member of the review team (J.P.S.) if necessary. Studies screened by title, abstract and full text were eligible for inclusion if they met all of the following criteria:

1. BP measured in a community pharmacy;
2. Pharmacy BP readings compared with any of ABPM, HBPM or general practitioner clinic BP readings in the same patients;
3. Participants aged 18 years or older.

Studies were excluded if any of the following applied:

1. Pharmacy readings were not compared with ABPM, HBPM or general practitioner clinic readings.
2. Participants had their BP measured in a pharmacy as part of a wider intervention or package of care to reduce or control BP – for example, patients who received more regular monitoring, or condition or medication advice over participants receiving usual care.
3. Patients who had atrial fibrillation or were pregnant.
4. Comparison clinic BP readings reported from secondary care settings.

### Outcomes measured

The primary outcome of this review was to compare the weighted mean SBP difference between community pharmacy and ABPM readings, measured as the difference between published estimates of mean CPBP and ABPM. Secondary outcomes explored the mean differences between SBP measured in the pharmacy, HBPM readings and general practitioner clinic readings, as well as corresponding diastolic comparison.

### Data extraction

Data were independently extracted and verified by two members of the review team (A.A. and J.O.S.). Data on mean BP values and SD, number of visits to the relevant setting, number of measurements per visit, the BP monitor used, patient age and sex, medication history and hypertensive status were extracted where present.

Where data were not available from the published articles, authors were contacted and the outstanding data requested. Discrepancies in data extraction were discussed and resolved with a third reviewer (J.P.S.) if necessary.

### Quality assessment

Included articles were independently assessed for quality at study level by two members of the review team (A.A. and J.O.S.). The Quality Assessment of Comparative Diagnostic Accuracy Studies-2 checklist was used to assess methodological quality [18]. Risk of bias and applicability concerns for patient selection, index test and reference test conduction, as well as flow and timing of patients through the studies were considered. Studies were considered to have a low risk of bias if:

1. They used consecutive or random sampling methods.
2. Index test results were interpreted without knowledge of the reference standard results and vice versa.
3. All patients received the same reference test.

The impact of studies of a lower quality, according to author consensus, was assessed in sensitivity analyses and the influence of individual studies on the overall summary estimate was assessed using an influence analysis.

### Data synthesis

Data were analyzed using a random effects meta-analysis of the weighted mean differences (WMDs) between measurement modalities using STATA statistical software (StataCorp 2011, Release 12; StataCorp, College Station, Texas, USA). A random-effects model was used to account for expected heterogeneity in baseline population characteristics, sample sizes and BP measurement protocols between studies [19].

Mean BP and SDs from relevant studies were synthesized comparing: pharmacy and daytime ABPM (primary outcome), as well as pharmacy and 24-h ABPM, pharmacy and HBPM and pharmacy and general practitioner clinic. A-priori subgroup analyses taking into account study quality and baseline hypertensive status were conducted on all comparisons where possible.

All data are presented as proportions of each study population, means with SD or WMD with 95% CIs (confidence intervals) unless otherwise stated.

## RESULTS

### Study selection

After removal of 677 duplicate records, a total of 3138 unique studies were identified from the literature searches. An additional three records were found from citation searches. Two thousand, five hundred and seventy-seven studies were excluded at title screening and 534 studies were excluded after abstract screening, resulting in 30 studies eligible for full-text review.

**FIGURE 1:**
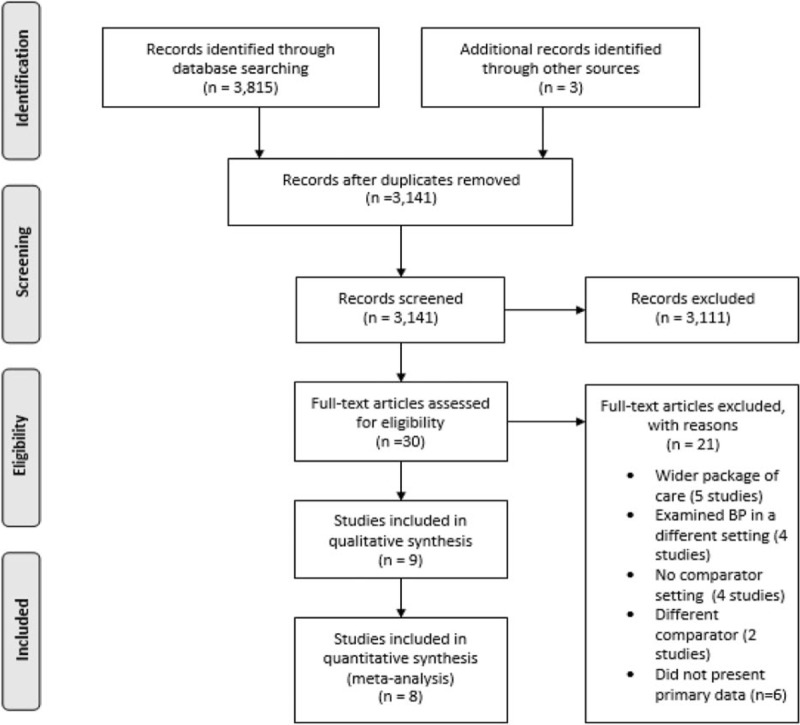
Preferred Reporting Items for Systematic reviews and Meta-Analyses flow diagram showing selection of studies included in this review.

Twenty-one articles screened at the full-text stage were excluded because they either: examined CPBP as part of a wider package of care, such as incorporating measurements during interventions to reduce BP (*n* = 5); examined the BP measurements in a different setting, such as pharmacy outpatient clinics (*n* = 4); presented no comparator setting (*n* = 4); used a different comparator (e.g. compared pharmacy readings with secondary care BP readings, *n* = 2); or did not present primary data (*n* = 6). In total, nine studies were eligible for inclusion in the review, eight of which were included in the data-synthesis (Fig. 1). One eligible study [18] could not be included in the meta-analysis as no data relating to BP readings taken in a pharmacy or home setting were presented, despite reporting conducting these measurements in their methods. The corresponding author was contacted on three occasions without reply.

### Study characteristics

A total of three studies compared CPBP and daytime ABPM readings (*n* = 319), five studies compared CPBP and HBPM readings (*n* = 1848) and six studies compared CPBP and general practitioner clinic readings (*n* = 2100) (Table 1). Studies were conducted in Spain (*n* = 4), Canada (*n* = 2), Turkey (*n* = 1) and Switzerland (*n* = 1). The mean age of study participants included in the quantitative synthesis was 58.6 years and mean proportion of women 53.5%. Four studies included only hypertensive patients [15,16,20,21], whereas four other studies recruited a mixed cohort of hypertensive and normotensive individuals [22–25]. One study did not specify the baseline BP status of participants [26].

**TABLE 1:** Study characteristics

Reference	Sample size	Mean systolic CPBP [mmHg (SD)]	Mean age [years (SD where reported)]	Population	Measurement modalities compared	% female	Location
Divisón *et al.* [26]	96	131.4 (24.5)	57.3	Not stated	CP, GP clinic	63	Spain
Botomino *et al.* [22]	50	129.0 (19.0)	53.7 (14.0)	Mixed	CP, ABPM, HBPM	58	Switzerland
Aleman *et al.* [20]	1399	157.0 (13.4)	60.1 (9.7)	Hypertensive	CP, HBPM, GP clinic	50	Spain
Sabater-Hernandez *et al.* [15]	169	128.3 (14.7)	56.4 (10.6)	Hypertensive	CP, ABPM, HBPM	60	Spain
Sendra-Lillo *et at.* [16]	70	128.0 (14.8)	61.8 (12.4)	Hypertensive	CP, HBPM, GP clinic	44	Spain
Chambers *et al.* [23] (group A)	136	121.8 (14.1)	75.9 (6.5)	Mixed	CP, GP clinic	49	Canada
Chambers *et al.* [23] (group B)	139	127.6 (16.5)	75.9 (6.8)	Mixed	CP, GP clinic	53	Canada
Chung *et al.* [24]^a^	1838	Not stated	66.0 (10.0)	Mixed	CP, HBMP	62	Not stated
Padwal *et al.* [21]	100	137.8 (13.7)	59.7 (12.8)	Hypertensive	CP, ABPM, GP clinic	53	Canada
Mutlu *et al.* [25]	160	145.7 (12.9)	44.4 (15.3)	Mixed	CP, ABPM, HBPM and GP clinic	46	Turkey

ABPM, ambulatory blood pressure monitoring; BP, blood pressure; CP, community pharmacy; CPBP, community pharmacy blood pressure; GP, general practitioner; HBPM, home blood pressure monitoring.

^a^Study not included in quantitative synthesis due to lack of data presented.

BP readings taken in the pharmacy were conducted by a pharmacist (*n* = 5) [15,16,22,24,26], pharmacist/pharmacy technician (*n* = 2) [20,25] or via automated machine with no member of the pharmacy team (*n* = 2) (Table 2) [21,23]. Studies included in this review varied in pharmacy BP measurement protocols, varying in the number of visits to the pharmacy (one to five visits), the number of readings per visit (two to six readings) and the operator in each setting responsible for conducting the BP readings (pharmacist, pharmacy staff or unattended kiosk).

**TABLE 2:** Blood pressure measuring methods

Reference	Setting	No. readings per visit	No. of visits	Protocol for calculating mean	Measured by
Divisón *et al.* [26]	Pharmacy	3	1	Mean of three readings	Pharmacist
	GP clinic	3	1	Mean of three readings	Nurse
Botomino *et al.* [22]	Pharmacy	2	1	Mean of two readings	Pharmacist
	ABPM	54	1 day	Mean of daytime readings	Automatic
	HBPM	4	4 days	Mean of days 2–4	Patient
Aleman *et al.* [20]	Pharmacy	2	3	Mean of two readings per visit	Pharmacist or technician
	HBPM	3	3 mornings	Mean of all readings per sitting	Patient
	GP	2	3	Mean of two readings per visit	Patient
Sabater-Hernandez *et al.* [15]	Pharmacy	3	4	Discarded first reading from each visit, mean over all four visits	Pharmacist
	ABPM	45	1 day	Mean daytime and 24-h readings	Automated
	HBPM	6	4 days	Discarded day 1 readings and first readings each morning and evening	Patient
Sendra-Lillo *et at.* [16]	Pharmacy	3	5	Mean of all readings from first three visits	Pharmacist
	HBPM	6	4 days	Mean of days 2–4	Patient
	GP clinic	3	3	Mean of all readings	GP and nurse
Chambers *et al.* [23]	Pharmacy	6	1	Mean of readings two to six	Automated
	GP clinic	6	1	Mean of readings two to six	Automated
^a^Chung *et al.* [24]	Pharmacy	3	4	Mean of last two measurements from all four visits	Pharmacist
	HBPM	6	12 days	Discarded day 1 readings and first morning and evening readings thereafter	Patient
Padwal *et al.* [21]	Pharmacy	3	4	Mean of each visit, as well as overall mean	Automated (kiosk)
	ABPM	45	1 day	Mean daytime and 24-h readings	Automatic
	GP clinic	3	1	All readings	Research assistant
Mutlu *et al.* [25]	Pharmacy	Not stated	1	Not stated	Pharmacy employees
	ABPM	Not stated	1 day	Mean of 24-h readings	Patient
	HBPM	Not stated	1 day	Not stated	Patient
	GP clinic	Not stated	2	Not stated	GP

ABPM, ambulatory blood pressure monitoring; GP, general practitioner; HBPM, home blood pressure monitoring.

^a^Not included in quantitative synthesis due to lack of data.

### Quality assessment

Risk of bias for each domain in each included study is presented in Table 3. All studies had some degree of bias or lack of clarity in methodological reporting.

**TABLE 3:** QUADAS-2 assessment of study quality

	Risk of bias				Applicability concerns			
Reference	Patient selection	Index test	Reference standard	Flow and timing	Patient selection	Index test	Reference standard	Overall risk of bias
Divisón *et al.* [26]	High	High	High	Unclear	High	High	Unclear	High
Botomino *et al.* [22]	High	Unclear	Unclear	Low	High	High	Low	High
Aleman *et al.* [20]	Unclear	Unclear	High	Low	High	Low	Low	Unclear
Sabater-Hernandez *et al.* [15]	Low	Unclear	Low	Unclear	Low	Low	Low	Low
Sendra-Lillo *et al.* [16]	Low	Unclear	Unclear	Unclear	Low	Low	Low	Low
Chambers *et al.* [23]	High	High	High	Low	Unclear	Low	High	High
^a^Chung *et al.* [24]	Unclear	Low	Unclear	Low	Low	Low	Low	Low
Padwal *et al.* [21]	High	Unclear	Low	Low	Low	Low	Low	Low
Mutlu *et al.* [25]	High	Unclear	Low	Low	High	Low	Low	High

^a^Not included in quantitative synthesis due to lack of data.

All but two studies [23,26] used a suitable reference standard to accurately estimate patient's out-of-office BP (i.e. ABPM or HBPM). The timing of index and reference measurements, with respect to each other, was generally poorly reported. Seven out of eight studies included in the meta-analysis used internationally validated BP monitors, with the other using a nonvalidated wrist device [25].

**FIGURE 2:**
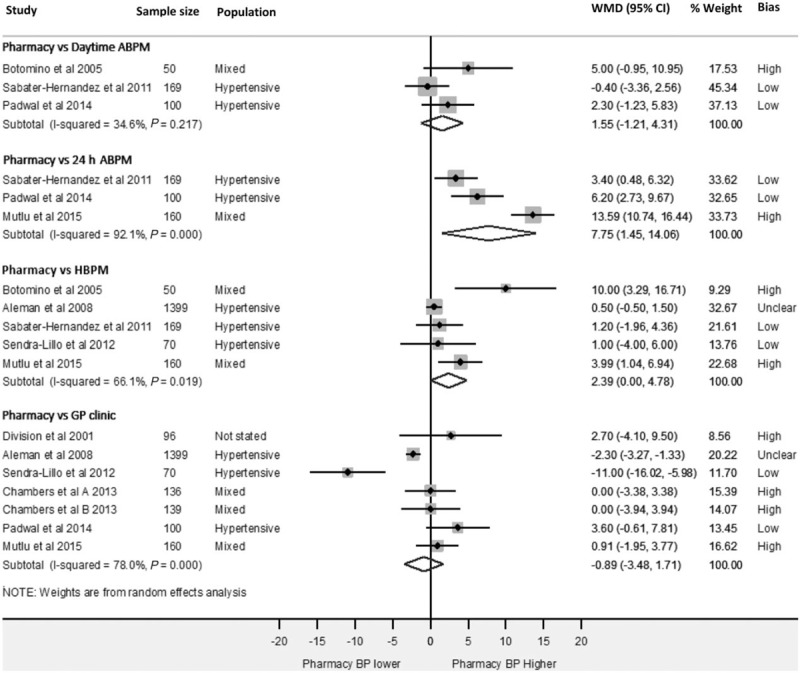
Forest-plot showing weighted mean SBP differences between; pharmacy and daytime ambulatory blood pressure monitoring, pharmacy and 24-h ambulatory blood pressure monitoring, pharmacy and home and pharmacy and general practitioner clinic readings. ABPM, ambulatory blood pressure monitoring; Bias, risk of bias; GP, general practitioner; HBPM, home blood pressure monitoring; WMD, weighted mean difference.

As none of the comparisons in Fig. 2 included 10 or more studies, the extent of publication bias could not be reliably assessed [27].

### Pharmacy and daytime ambulatory blood pressure readings

Three studies (*n* = 319, mean age 57.0 years) reported extractable data comparing CPBP and daytime ABPM readings (Fig. 2) [15,21,22]. Daytime readings were defined inconsistently in the included studies, with two studies using readings from 0700 to 2200 h [15,21], and one study using measurements from 0600 to 1800 h [22]. All studies included more than the minimum of 14 daytime (0700–2300 h) ABPM readings required by UK guidelines [5,9]. Overall, pooled analysis of CPBP and daytime ABPM readings (Fig. 2) showed no significant difference between the two measurement modalities [WMD 1.6 mmHg (95%CI −1.2 to 4.3), statistical heterogeneity *I*^2^ = 34.6%, *P* = 0.22].

### Pharmacy and 24-h ambulatory blood pressure readings

Three studies [15,21,25] (*n* = 429, mean age 52.7 years) reported a comparison of CPBP and 24-h ABPM (Fig. 2). All readings were from a single 24-h period. Mean BP recorded in pharmacies were significantly higher than 24-h ABPM readings [WMD 7.8 mmHg (95% CI 1.5–14.1)]. However, there was evidence of considerable statistical heterogeneity between included studies (*I*^2^ = 92.1%, *P* < 0.001) [28].

### Pharmacy and home blood pressure readings

Five studies [15,16,20,22,25] compared mean systolic CPBP readings with HBPM (*n* = 1848, mean age 58.3 years) (Fig. 2). A sixth study stated that a CPBP–HBPM comparison was made but did not report this data in their analysis [24]. Mean SBP in pharmacy was 2.4 mmHg (95% CI 0.0–4.8) higher than HBPM readings, with differences ranging from 0.5 to 10.0 mmHg between studies. The observed statistical heterogeneity was significant (*I*^2^ = 66.1%, *P* = 0.02).

### Pharmacy and general practitioner clinic blood pressure readings

Across the six studies comparing mean systolic CPBP to general practitioner clinic measurements (*n* = 2100, mean age 60.9 years) [16,20,21,23,25,26], there was no evidence of a difference between the two measurement modalities [WMD −0.9 mmHg (95% CI −3.5 to 1.7)] (Fig. 2). One study reported findings from two separate cohorts of patients and so is included separately in the meta-analysis as Chambers *et al.*[23] group A and Chambers *et al.*[23] group B. Mean differences between studies ranged from −2.3 (pharmacy readings lower than general practitioner) to 11.0 mmHg (pharmacy readings higher than general practitioner), and there was evidence of considerable statistical heterogeneity between included studies (*I*^2^ = 78.0%, *P* < 0.001). Of the 2100 patients in this comparison, 1399 (67%) were from one study [20], whose results suggest pharmacy readings to be significantly lower than general practitioner clinic measurements [WMD −2.30 mmHg (95% CI −3.27 to −1.33)].

### Diastolic readings

Corresponding DBP comparisons (Fig. 4) show CPBP to be significantly higher than both daytime ABPM [WMD 2.96 mmHg (95% CI 1.27–4.64)] and 24-h ABPM [WMD 6.52 mmHg (95% CI 5.19–7.85)]. There was no significant difference between CPBP and HBPM [WMD 1.61 mmHg (95% CI −0.63 to 3.84)] or between CPBP and general practitioner clinic readings [WMD −0.24 mmHg (95% CI −1.50 to 1.02)].

### Subgroup analyses

**FIGURE 3:**
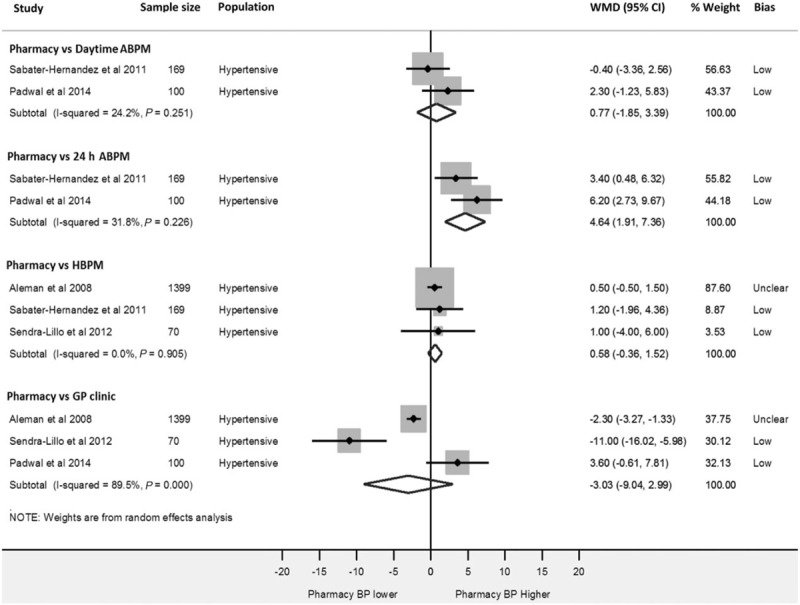
Sensitivity analysis. Forest-plot showing weighted mean SBP differences between; pharmacy and daytime ambulatory blood pressure monitoring, pharmacy and 24-h ambulatory blood pressure monitoring, pharmacy and home and pharmacy and general practitioner clinic readings after removal of studies exhibiting ‘high risk’ of bias. ABPM, ambulatory blood pressure monitoring; Bias, risk of bias; GP, general practitioner; HBPM, home blood pressure monitoring; WMD, weighted mean difference.

The influence of studies considered to exhibit high risk of bias was explored through sensitivity analyses. Figure 3 shows pooled estimates of all comparisons after removal of ‘high risk’ of bias studies. As with the primary analyses, these results show a nonsignificant difference between CPBP and daytime ABPM readings [WMD 0.8 mmHg (95% CI −1.9 to 3.4)] and CPBP and HBPM [WMD 0.6 mmHg (−0.4 to 1.5)]. Heterogeneity was reduced for the 24-h ABPM (92–32%) and HBPM comparisons (66–0%). CPBP remained significantly higher than 24-h ABPM readings. Removal of ‘high-risk’ studies narrowed confidence intervals across CPBP and daytime ABPM, 24-h ABPB and HBPM. The interpretation of CPBP in relation to general practitioner clinic readings remained unchanged by this analysis, with no difference observed.

Further subgroup analysis, accounting for baseline hypertensive status (hypertensive or mixed cohorts) across all four modality comparisons did not significantly alter the study findings. Influence analyses, whereby each study is omitted in turn within each comparison did not significantly alter the interpretation of the summary estimates (Supplementary Appendix 3), suggesting that no one study had undue influence on the summary estimates above all others.

## DISCUSSION

### Summary of findings

The current systematic review identified all existing literature comparing BP measurements in community pharmacies with ambulatory, home and general practitioner clinic measurements in the same patients. Pooled summary estimates from eight studies included 2319 patients. The evidence suggested no significant difference between systolic CPBP and daytime ABPM, and this result was unchanged by the removal of studies exhibiting high risk of bias. CPBP was significantly higher, both statistically and clinically, than 24-h ABPM readings [+7.8 mmHg (95% CI 1.5–14.1)]. As 24-h ABPM readings include nighttime measurements, and the associated night-time BP dip, it is unsurprising that the pharmacy–24-h ABPM comparison showed this.

The comparison between CPBP and general practitioner readings was inconclusive, with no evidence of a difference between measurement modalities but high heterogeneity. In the case of home BP, the primary analysis showed a significant CPBP–HBPM difference, but this was not observed in the sensitivity analysis excluding low-quality studies (Fig. 3), suggesting that further work is required to accurately define the relationship between CPBP and HBPM.

**FIGURE 4:**
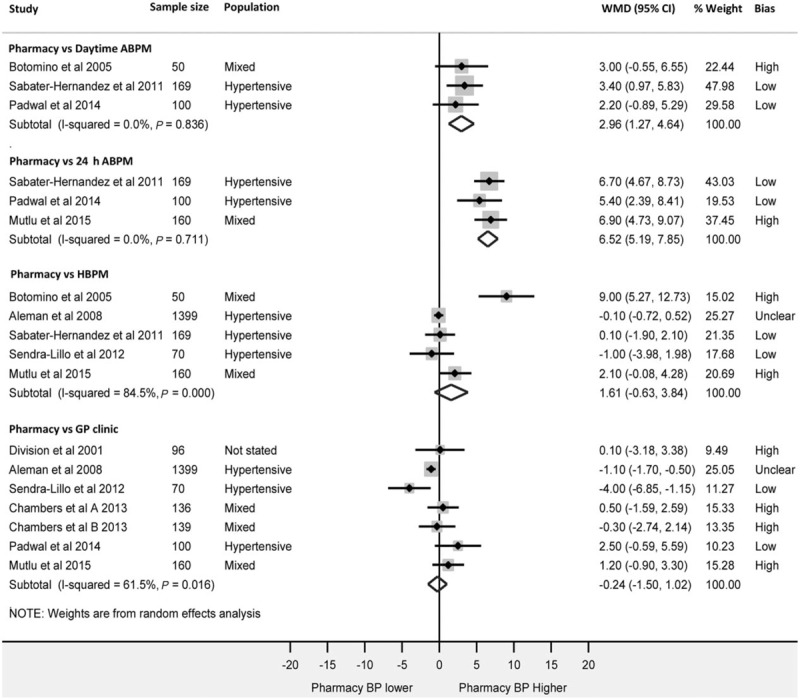
Forest-plot showing weighted mean diastolic blood pressure differences between; pharmacy and daytime ambulatory blood pressure monitoring, pharmacy and 24-h ambulatory blood pressure monitoring, pharmacy and home and pharmacy and general practitioner clinic readings. ABPM, ambulatory blood pressure monitoring; Bias, risk of bias; GP, general practitioner; HBPM, home blood pressure monitoring; WMD, weighted mean difference.

Despite DBP comparisons mirroring the SBP comparisons between CPBP and 24-h ABPM, HBPM and general practitioner clinic readings, the data presented in Fig. 4 suggests diastolic CPBP is significantly higher than daytime ABPM readings. Given that systolic readings are the main focus of clinical decision-making in primary care due to closer correlation with cardiovascular endpoints [29,30], the conclusions of this study are unchanged by this analysis.

Currently, BP readings taken in community pharmacies result in either referral to the general practitioner or advice to the patient, rather than formal diagnosis or regular patient follow-ups. These recommendations are based on the 140/90 mmHg threshold [13]. Given this context and the inconclusive nature of the data from primary studies, the presented evidence suggests that CPBP readings may be best interpreted using the daytime ABPM threshold for the diagnosis and management of hypertension (135/85 mmHg). This cautious approach would likely result in a higher sensitivity for detecting hypertension when referring patients to their general practitioner with borderline elevated BP, albeit at the expense of specificity. This could increase general practitioner workload through inappropriate referral; however, general practitioner referrals could be reduced by pharmacies referring patients for HBPM or daytime ABPM directly, to further refine referral criteria.

### Strengths and limitations

To our knowledge, this is the first meta-analysis comparing CPBP readings to guideline recommended BP measurement techniques. Our comprehensive search strategy included studies from diverse settings and countries, and meant that we were unlikely to have missed any relevant articles.

The interpretation of mean BP differences in this review was affected by inconsistent methodology between included studies, particularly in the measurement of CPBP. Four [22,23,25,26] of the eight studies calculated mean pharmacy BP from one visit, whereas all other studies used three or more visits. One study measured pharmacy and general practitioner clinic BP using a nonvalidated wrist monitor [25]. Studies where mean CPBP was calculated from eight [15] or 12 [21] BP readings over several pharmacy visits correlated more closely with daytime ABPM than studies where CPBP was calculated from two readings at a single visit [22]. Importantly, however, readings from a single pharmacy visit may be most closely related to current BP measurement protocols in pharmacies [13]. Furthermore, in two studies comparing CPBP with general practitioner clinic BP [16,26], nurses were involved in clinic BP measurements rather than a general practitioner. A systematic review comparing nurse with general practitioner measured BP, has estimated that BP taken by a nurse can be up to 7 mmHg lower on average than general practitioner measured readings [31]. A further study used a research assistant to measure clinic BP, in which an automated device was used with the patient left alone for the duration of the measurement period [21].

These variations in measurement protocols are the likely cause of the inconsistent results we have found and are likely to have contributed to the heterogeneity observed in our analyses.

The largest study eligible for inclusion (*n* = 1838) [24] did not publish extractable BP data for pharmacy and home readings, nor were we able to contact the authors. It is unclear how inclusion of this study could have affected the ‘pharmacy vs. home’ comparison [+2.4 mmHg (95% CI 0.0–4.8)] or the heterogeneity in that comparison.

Where no difference was seen between measurement modalities, the issue of whether sufficient statistical power was available to detect a true difference should be considered. Three studies compared CPBP with daytime ABPM and only two of these presented a sample size calculation to adequately power their study [15,21], with only one study recruiting a sufficient number of participants to meet their power calculation target [21]. Although pooling data from multiple, similar, studies as part of a meta-analysis has the advantage of increasing the power to detect a true difference in BP between measurement modalities, it is still possible that even with these additional data, the comparisons may have remained underpowered.

Furthermore, it was not possible to extract mean differences of individual patients from the articles in this review, resulting in mean differences between measurement modalities to be estimated from the overall mean BP data presented in each study. This method results in larger standard errors, and thus, wider confidence intervals than if individual patient mean differences were available. As such, the findings of this review are likely to be conservative in their estimations of how CPBP compares with other measurement modalities.

### Comparison with previous literature

One previous systematic review conducted in 2009 [14] compared pharmacy BP readings with ABPM, HBPM and general practitioner clinic readings and found three studies but was unable to conduct a meta-analysis due to lack of data and concerns regarding heterogeneity. The current review presents the pooled data from a further five studies (i.e. eight in all) combined in a random effects meta-analysis, due to remaining heterogeneity between studies.

A 2011 systematic review by Hodgkinson *et al.*[32] compared the diagnostic accuracy of home and general practitioner clinic BP readings with that of ABPM readings. This meta-analysis included two studies comparing HBPM with ABPM, six comparing general practitioner clinic BP with ABPM and one study comparing all three measurement methods. Their overall findings suggested neither clinic nor home measurement could be recommended as a single diagnostic test and therefore, recommended that clinic readings should not be relied upon alone for the diagnosis of hypertension without ABPM measurements as confirmation. For the diagnostic accuracy of CPBP measurements to be ascertained, a receiver operating characteristic curve of its sensitivity and specificity for diagnosing hypertension would be required, and although sought, these data were not sufficiently reported in the primary studies in this review.

### Implications for clinical practice

Findings from this meta-analysis suggest that CPBP may be best interpreted using daytime ABPM thresholds for hypertension diagnosis and management. In the absence of additional high-quality and adequately powered studies, using the 135/85 mmHg threshold for hypertension is likely to ensure a higher sensitivity for detecting and referring patients with true underlying hypertension to their general practitioner or for out-of-office measurements. For instance, if a patient's CPBP was measured at 138/85 mmHg, using this threshold would likely result in the patient being advised to see their general practitioner, encouraged to monitor their BP at home or be given healthy lifestyle advice. If however the 140 mmHg threshold was employed, this patient would be less likely to get such advice. The former approach is more conservative but would likely benefit more patients whose clinic and out-of-office measurements would later suggest a persistently high BP. The presented data does not, however, support the utilization of CPBP as an alternative to current out-of-office BP measures.

BP checks delivered in community pharmacies can be more convenient to patients as no appointments are generally required [33] and services can be delivered at lower cost compared with other overstretched primary care settings such as general practitioner clinics [34]. A better understanding of how CPBP readings compare with those taken in a traditional clinic environment as well as ABPM and HBPM will assist in the clinical interpretation of CPBP readings and may support future nationally commissioned BP screening or management services in pharmacies.

Further work should explore the most effective referral strategy for patients with raised CPBP and the cost effectiveness of these strategies compared with traditional methods of screening.

### Conclusion and recommendations

Studies comparing mean CPBP with general practitioner clinic and out-of-office measurements of BP were generally small, underpowered and exhibited important methodological differences in the way mean BP was estimated. Given the limitations of these studies and the role community pharmacies play in the diagnosis and management of hypertension, CPBP readings should be cautiously interpreted using the daytime ABPM threshold for the diagnosis and management of hypertension (i.e. 135/85 mmHg), until more adequately powered studies become available.

## ACKNOWLEDGEMENTS

We would like to thank Dr Thanusha Ananthakumar for her help with title screening at the start of this review and Mr Pablo Muñoz Rodríguez for his help translating some of the Spanish articles included in this article.

The current systematic review was carried out as part of a DPhil scholarship awarded to A.A. funded by the Primary Care Research Trust, The University of Oxford and NIHR Oxford CLAHRC. J.P.S. was funded by a Medical Research Council Strategic Skills Postdoctoral Fellowship (MR/K022032/1) and now receives support from the NIHR Oxford CLAHRC. R.J.M. holds an NIHR Professorship (NIHR-RP-R2-12-015) and receives support from the NIHR Oxford CLAHRC. J.O.S. holds a Clarendon Scholarship.

This article presents independent research funded by the NIHR. The views expressed are those of the authors and not necessarily those of the NHS, the NIHR or the Department of Health.

### Conflicts of interest

There are no conflicts of interest.

## Supplementary Material

Supplemental Digital Content

## Reviewers’ Summary Evaluations

### Reviewer 2

This is a useful meta-analysis demonstrating that mean community pharmacy BP measurements are quite comparable to ABPM and home BP measurements. Community pharmacies are highly accessible and ideally positioned for population-based screening purposes. In some jurisdictions, in-pharmacy BP kiosks are available, with measurements integrated into the electronic record. Device quality can vary between pharmacies; thus, it is important to ensure that only validated devices are used.
